# Tissue-Specific Contributions of Paternally Expressed Gene 3 in Lactation and Maternal Care of *Mus musculus*

**DOI:** 10.1371/journal.pone.0144459

**Published:** 2015-12-07

**Authors:** Wesley D. Frey, Joomyeong Kim

**Affiliations:** Department of Biological Sciences, Louisiana State University, Baton Rouge, Louisiana, 70803, United States of America; Florida State University, UNITED STATES

## Abstract

Paternally Expressed Gene 3 (*Peg3*) is an imprinted gene that controls milk letdown and maternal-caring behaviors. In this study, a conditional knockout allele has been developed in *Mus musculus* to further characterize these known functions of *Peg3* in a tissue-specific manner. The mutant line was first crossed with a germline Cre. The progeny of this cross displayed growth retardation phenotypes. This is consistent with those seen in the previous mutant lines of *Peg3*, confirming the usefulness of the new mutant allele. The mutant line was subsequently crossed individually with MMTV- and Nkx2.1-Cre lines to test *Peg3*’s roles in the mammary gland and hypothalamus, respectively. According to the results, the milk letdown process was impaired in the nursing females with the *Peg3* mutation in the mammary gland, but not in the hypothalamus. This suggests that *Peg3*’s roles in the milk letdown process are more critical in the mammary gland than in the hypothalamus. In contrast, one of the maternal-caring behaviors, nest-building, was interrupted in the females with the mutation in both MMTV- and Nkx2.1-driven lines. Overall, this is the first study to introduce a conditional knockout allele of *Peg3* and to further dissect its contribution to mammalian reproduction in a tissue-specific manner.

## Introduction

Paternally Expressed Gene 3 (*Peg3*) is an imprinted gene located on human chromosome 19q13.4 and mouse chromosome 7qA1 [1,2]. *Peg3* encodes a protein with DNA-binding capabilities that is localized to the nucleus of neuronal cells [3,4]. Expression of *Peg3* is most dominant in the ovary, placenta and hypothalamus as observed in murine models [2,5]. The potential roles of *Peg3* in diseases ranging from low birth-weight to breast and cervical cancer have triggered the production of mutant mouse lines, which have been studied for multiple characteristics. The most striking and consistent characteristics observed in a previous *Peg3* knock-in line is a maternal caring problem in *Peg3*-deficient dams and a growth defect in neonatal pups [5,6,7]. More recently, our group has generated a mouse line with a deletion in the promoter region as well as a strain carrying a transcriptional truncation, which were both reported to have similar effects [8,9].

*Peg3*-deficient dams have also been linked to inadequate milk letdown as well as problems with maternal-caring behaviors [6]. However, the process of milk letdown is complex, requiring a stimulus and response from both the neonate and the mother in multiple organs. In response to a pup suckling on a dam's teat, the hypothalamus of the dam is triggered to release oxytocin into the bloodstream. Oxytocin then signals myoepithelial cells of the mammary to contract, releasing milk through ducts in the teat [10]. It has been shown that oxytocin release in milk ejection has a pro-social effect on the dam as well as a calming effect on the pup, tying the two systems in synchrony and producing positive reinforcement cues for the mother as well as the offspring [5,11].The neonatal growth, lactation and maternal care effects observed in *Peg3* mutant mouse lines are similar to the effects seen in oxytocin and oxytocin receptor-deficient mice in previous literature [12,13,14]. Furthermore, in *Peg3*-deficient mice, oxytocin neuron density is lower than wild-type littermates [6]. This suggests that *Peg3* may be acting upstream of the neuropeptide or the receptor. However, the neuropeptide/receptor interplay between organ systems is a circuit. Often, the shutdown of a single component in the system results in a more dramatic effect than shutting down the entire system. In that regard, we wanted to test the individual tissues responsible for the phenotypes observed in *Peg3*-deficient mice. To narrow down the systems responsible for the developmental, physiological and behavioral roles of *Peg3*, we generated and characterized a line that allows for conditional deletion of the gene *in vivo*. Using this conditional knockout line, we deleted *Peg3* in the hypothalamus and mammary gland to observe defects in neonatal growth, lactation and maternal care.

## Results

### Molecular characterization of *Peg3* ^FlpKO^ and *Peg3* ^DelKO^ mouse lines

**Fig 1:**
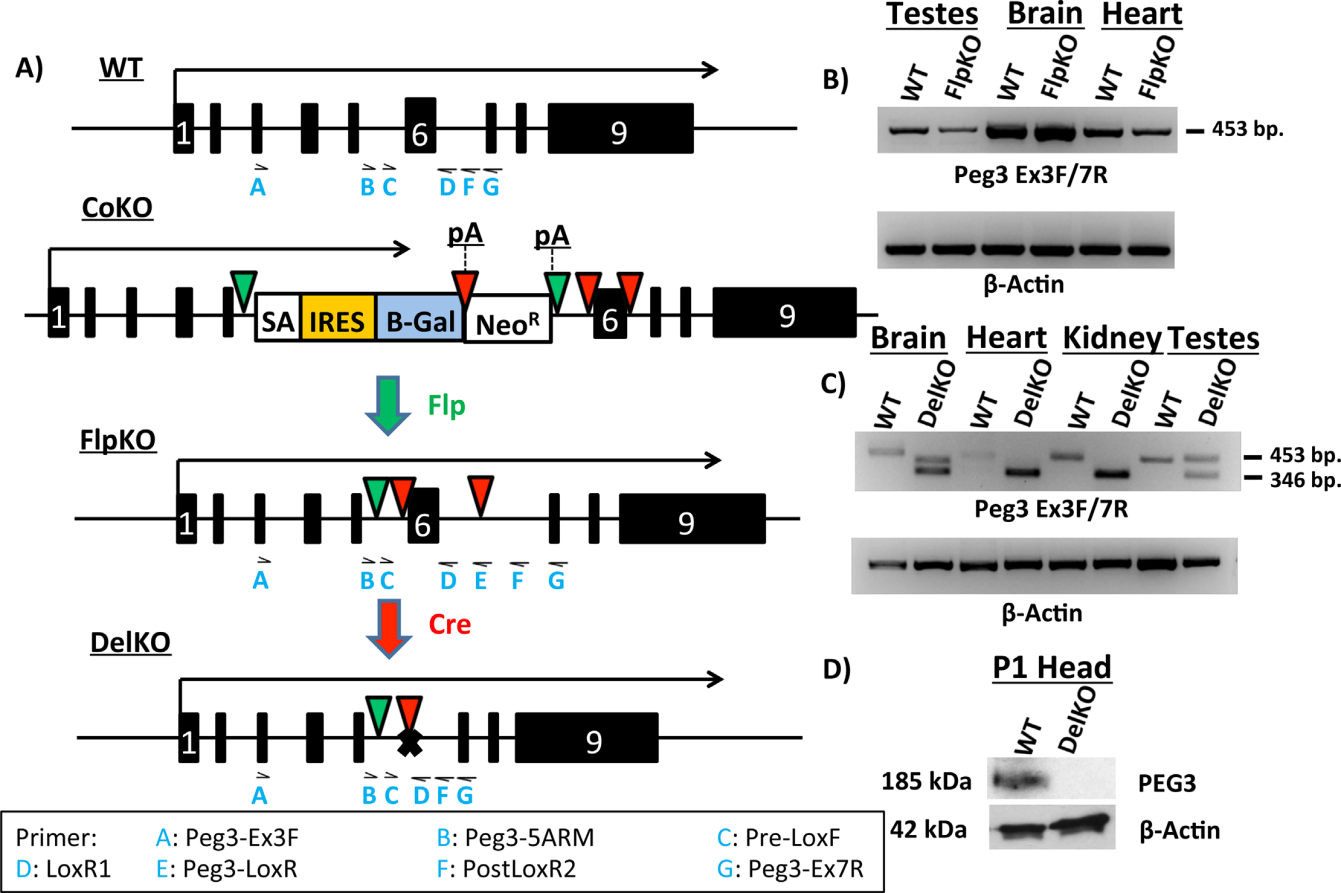
Molecular characterization of *Peg3* ^FlpKO^ and *Peg3* ^DelKO^ mouse lines. **(a)** Schematic representation of *Peg3* alleles. Arrows above each allele indicate transcriptional direction and length. Exons are indicated by boxes, with Exon 6 denoted as a white “6”. Flippase recognition target (FRT) sites are shown as green triangles. LoxP sites are indicated by red triangles. In the *Peg3* ^CoKO^ allele, we inserted a cassette containing a splice acceptor (SA) sequence, an internal ribosomal entry site (IRES) and a β-Galactosidase (β-Gal) reporter gene, followed by a poly-adenylation signal (pA). Neomycin Resistance gene (Neo^R^) is followed by another pA. Crossing *Peg3* ^CoKO^ mice with a Flp-expressing line results in the *Peg3* ^FlpKO^ allele. Successive crossing of *Peg3* ^FlpKO^ mice with Cre-expressing lines results in the *Peg3* ^DelKO^ allele. (**b)** RT-PCR of *Peg3* from various tissues in *Peg3* ^FlpKO^ line. β-Actin was used as an internal control. (**c)** RT-PCR of *Peg3* from various tissues in *Peg3* ^DelKO^. Cre-mediated recombination of Exon 6 results in the smaller amplicon size (346 bp in length) as compared to the wild-type product (453 bp in length). (**d)** Western blots from the 1-day-old heads of *Peg3* ^DelKO^. To visualize expression of PEG3 protein, western blots were probed for *Peg3*, stripped and then probed for β-Actin. Locations of primers used in this study are indicated as blue letters under each allele. The Primer legend at the bottom shows which primer corresponds to each abbreviation.

In order to test when and where *Peg3* expression is required for proper growth and development, two new mouse lines have been generated, *Peg3* ^FlpKO^ and *Peg3* ^DelKO^ (**Fig 1A**). The first line was generated by recombination of a knock-in allele (*Peg3* ^CoKO^) of *Peg3* by Flp recombinase [10]. Upon recombination, two poly-adenylation sites were excised, recovering the full-length transcription of *Peg3* (*Peg3* ^FlpKO^). In this line, Exon 6 is flanked by two LoxP sites. This results in a conditional knockout-ready line, which can be bred with Cre-recombinase-expressing (Cre) lines to produce a deletion of Exon 6. The deletion of Exon 6 generates the second mouse line, *Peg3* ^DelKO^ (**Fig 1A**). This deletion causes a frameshift and subsequent translational truncation of the *Peg3* ORF (Open Reading Frame), which starts at Exon 3 and ends at Exon 9. We used three Cre-expressing lines in this study (Zp3-Cre, MMTV-Cre and Nkx2.1-Cre). Zp3-Cre was used to make the *Peg3* ^DelKO^ line through germline recombination, whereas MMTV and Nkx2.1-Cre were used for the tissue-specific deletion of Exon 6 in *Peg3*.

Proper splicing in the *Peg3* ^FlpKO^ line was first tested with RT-PCR using the total RNA isolated from multiple tissues of the wild-type (WT) and heterozygote (FlpKO) mice carrying the mutant allele paternally (**Fig 1B**). The results indicated no abnormal splicing between exons 3–7 of the *Peg3* ^FlpKO^ line based on detection of the same-size product (453 bp in length) between the WT and FlpKO samples (**Fig 1B**). Furthermore, a series of RT-PCR testing the different parts of the 9.0-kb transcript of *Peg3* also confirmed the full-length transcription of *Peg3* in the *Peg3* ^FlpKO^ line (**S2A Fig**) as well as the truncated *Peg3* ^DelKO^ transcript (**S5B Fig**). In the *Peg3* ^DelKO^ line, the proper expression of *Peg3* transcript lacking Exon 6 was also tested by RT-PCR in a similar scheme as the *Peg3* ^FlpKO^ line (**Fig 1C**). According to the results, RT-PCR generated the expected 346-bp product from the multiple tissues of *Peg3* ^DelKO^ line, which is lacking the 107-bp-long Exon 6. On the other hand, the same PCR generated the 453-bp product from WT tissues. However, the two tissues also produced additional products besides the 346-bp products. In the case of testis, the upper band (453 bp) corresponds to the product from the maternal allele due to its derepression in germ cells. In the case of the brain, sequencing of the 410-bp minor product revealed the presence of a small exon, 64 bp in length, between Exons 4 and 5. More detailed analysis with the total RNA from WT confirmed that this is a minor exon that is previously undetected but usually included as part of the normal transcript of *Peg3*. Proper protein expression in the *Peg3* ^DelKO^ line was tested by western blot using the protein extracts from the heads of one-day-old pups. The results indicated a complete abrogation of the protein Peg3, confirming that the deletion of Exon 6 causes the frameshift and subsequent truncation in the ORF of *Peg3* (**Fig 1D**). Taken together, these results confirm the successful generation of the *Peg3* ^FlpKO^ and *Peg3* ^DelKO^ mouse lines. Since the Cre lines used for the current study have the mixed background of C57BL/6J (B6) and 129/SvJ, the females from the *Peg3* ^FlpKO^ and *Peg3* ^DelKO^ lines have been backcrossed with B6 males for two years, thus deriving these lines with the relatively pure B6 background.

### Growth retardation in *Peg3* ^DelKO^ pups

**Fig 2:**
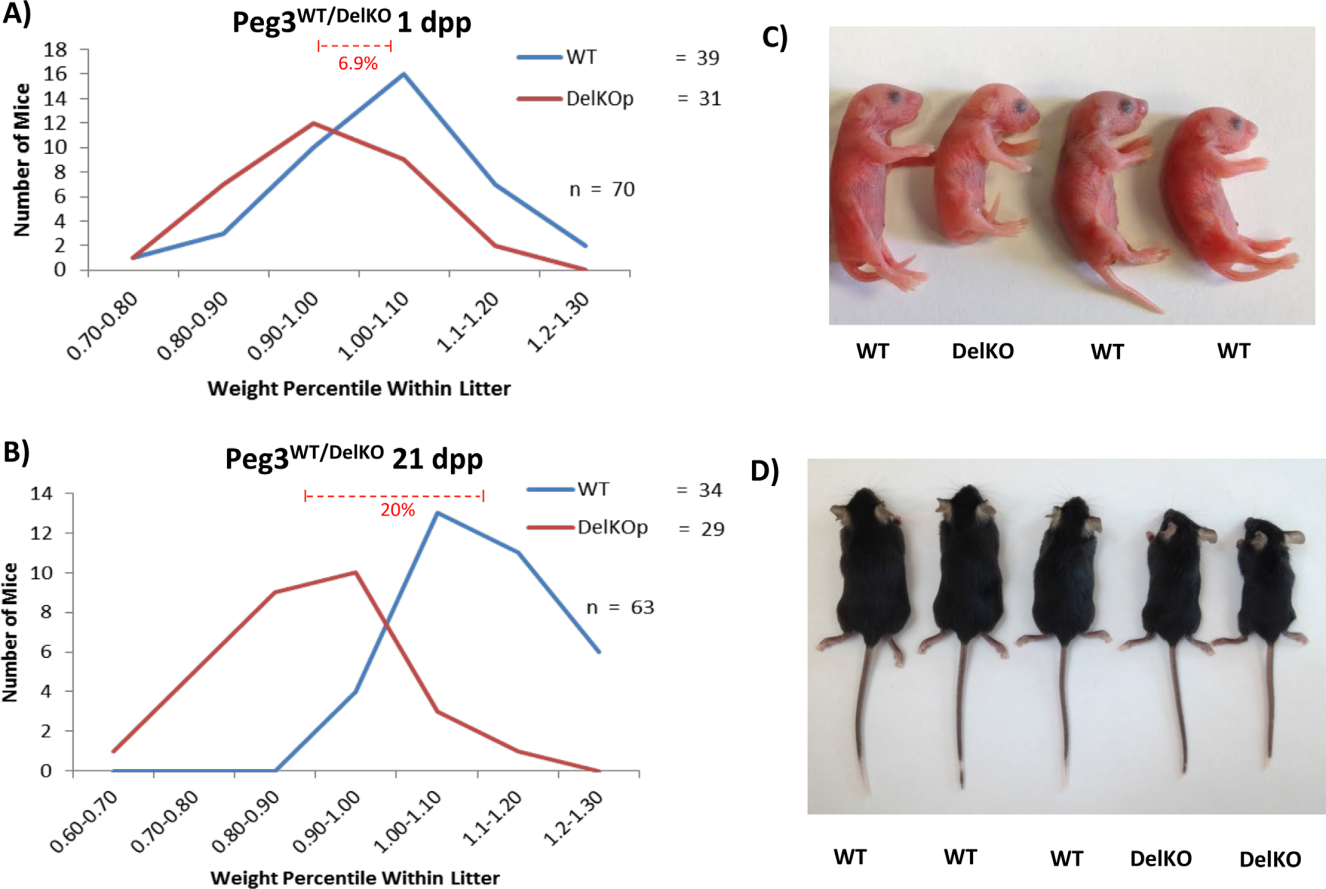
Growth effects of *Peg3* ^DelKO^ allele in neonates. **(a)** Male *Peg3* ^DelKO/WT^ were bred with female wild-type littermates to generate *Peg3* ^WT/DelKO^ pups with the paternal transmission of the mutant allele. Pups were genotyped and weighed at 1 day postpartum (dpp), then compared to the average weight of the litter. The number of pups belonging to each percentile weight range was then graphed to visualize the weight distribution of *Peg3* ^DelKO^ pups in comparison to their wild-type littermates. **(b)** The same series of analyses were also repeated with the pups at the weaning age (21 dpp). Representative littermates from 1 dpp and 21 dpp are shown with corresponding genotypes indicated (c,d).

To examine whether the *Peg3* ^DelKO^ line also displayed growth effects consistent with the previous studies [6,8,9], we performed breeding experiments measuring body weights at two different time points: 1 and 21 days postpartum (dpp). In these experiments, we crossed male *Peg3* ^DelKO/WT^ with C57BL/6J females. Their progeny were weighed and genotyped. Their weights were then converted into a percentile relative to the average weight of the litter. According to the results, at 1 dpp (**Fig 2A**), the average litter size was 8.5 pups, which varies marginally from the average litter size of B6 mice (~10 pups). The ratio between the heterozygous *Peg3* ^WT/DelKO^ pups and their *Peg3* ^WT/WT^ counterparts was 0.795:1, and did not vary significantly from the expected mendelian ratio (p = 0.3390). These results suggest that *Peg3* ^WT/DelKO^ mice had not been selected against during fertilization or during embryogenesis. The breeding results at 21 dpp (weaning age) also derived similar outcomes (**Fig 2B**). The average litter size was 9.0 pups and the transmission ratio was also similar to the expected mendelian ratio (*Peg3* ^WT/WT^:*Peg3* ^WT/DelKO^ = 0.85:1), again indicating that no selection had occurred against the *Peg3* ^WT/DelKO^ pups during the postnatal stages (p = 0.5287).

Although significant levels of lethality were not observed in the *Peg3* ^WT/DelKO^ pups, major growth retardation was observed at both 1- and 21-dpp time points (**Fig 2C and 2D**). At 1 dpp, the average weight difference was 6.99% between *Peg3* ^WT/DelKO^ pups and their WT littermates (p = 0.0028) (**Fig 2A**). A more dramatic growth effect was observed at 21 dpp, which displayed 20% weight difference between *Peg3* ^WT/DelKO^ pups and their WT littermates (p = 0.0001) (**Fig 2B**). Taken together, these results confirm the critical role of *Peg3* in controlling growth rates. The results also highlight a more significant role of *Peg3* during postnatal stages than during gestation in the newly derived *Peg3* ^WT/DelKO^ mutant line.

### *Peg3* conditional knockout pups: growth effects

The floxed allele *Peg3* ^WT/FlpKO^ was crossed with two Cre lines to test *Peg3*’s tissue-specific roles in controlling growth rates (**S2 Fig**). MMTV-Cre and Nkx2.1-Cre lines were employed to abolish *Peg3* expression in the mammary gland and hypothalamus, respectively. Recombination in these target organs was verified by PCR with primers flanking the inserted cassette **(S3 Fig)**. This series of breeding experiments were also analyzed in a similar manner as the *Peg3* ^DelKO^ line (described above), measuring the average litter size and body weights. For simplicity, the genotypes of the pups were abbreviated in the following manner: MMTV-Cre^tg/+^; *Peg3* ^WT/FlpKO^ as MMTV-Cre/FlpKO and MMTV-Cre^tg/+^; *Peg3* ^WT/WT^ as MMTV-Cre/WT.

**Fig 3:**
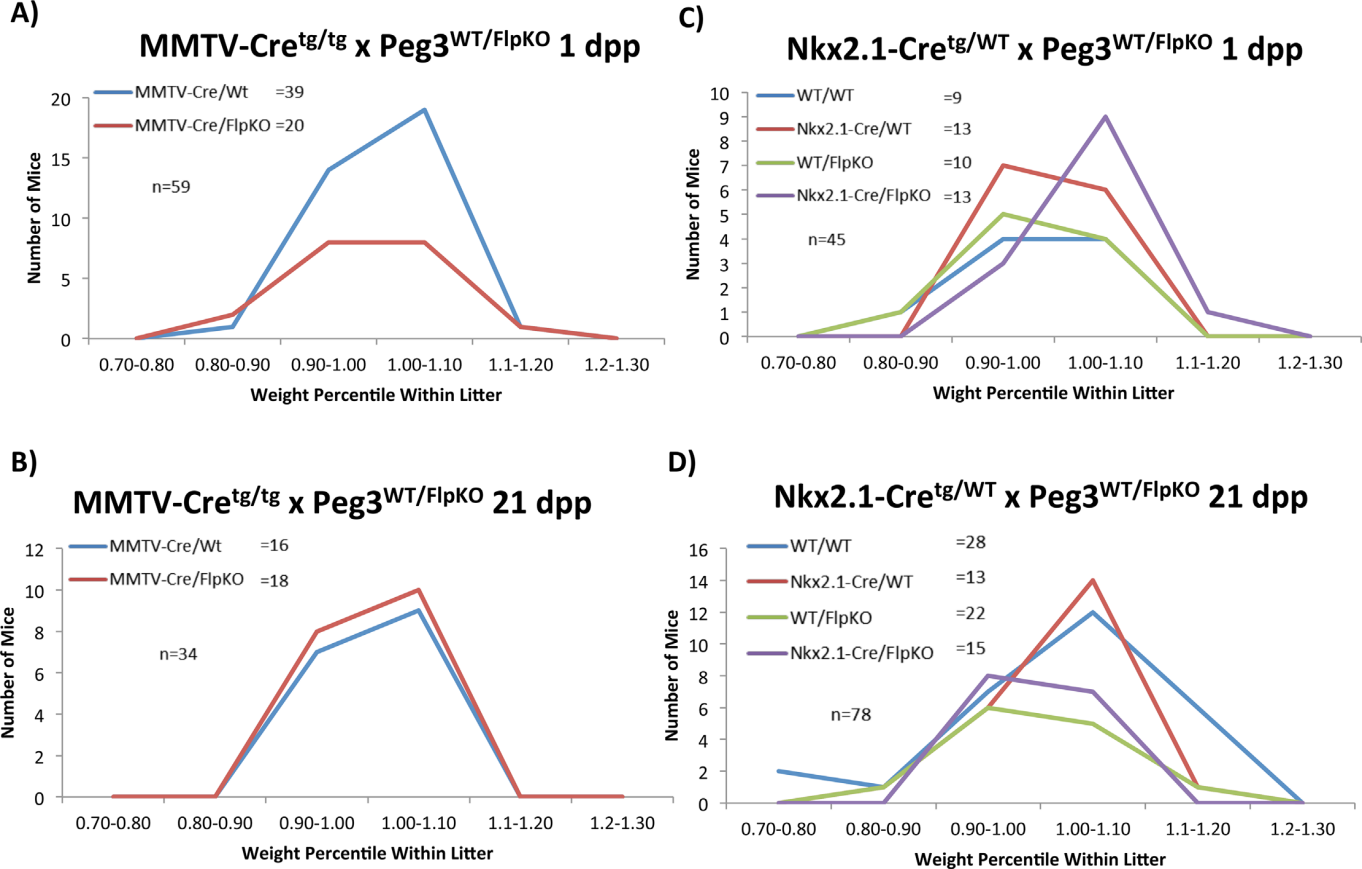
*Peg3* conditional knockout pups: impact on growth. Pups with conditional *Peg3* knockouts were weighed and compared to the average weight within the litter. Percentile weights within litter were then separated into 10% ranges. The numbers of pups in each percentile range are shown for MMTV-driven deletion at 1 dpp **(a)** and 21 dpp **(b)** and for Nkx2.1-driven deletion at 1 dpp **(c)** and 21 dpp **(d)**.

The breeding of male *Peg3* ^FlpKO/WT^ with female MMTV-Cre^tg/tg^ yielded the following results: At 1 dpp, the average litter size was 7.4 (59 pups/8 litters), which was slightly smaller than expected. The ratio between MMTV-Cre/WT and MMTV-Cre/FlpKO (39:20) deviated significantly from the expected mendelian ratio (1:1), suggesting that potential embryonic lethality is associated with MMTV-Cre/FlpKO pups (p = 0.0134). This embryonic lethality may be attributed to the small first litter size in MMTV-Cre mothers or expression of MMTV-Cre in the embryo-driven placenta, assumed by the presence of recombination **(S3 Fig)**. At 21 dpp, the average litter size was 6.8 (34 pups/5 litters) and the ratio of MMTV-Cre/WT and MMTV-Cre/FlpKO (16:18) was close to the mendelian ratio (p = 0.7317). This indicates that no obvious lethality is associated with the MMTV-Cre/FlpKO pups at the 21-dpp time point. The weight profiles also show no major difference between MMTV-Cre/WT and MMTV-Cre/FlpKO at both 1 and 21-dpp time points (p = 0.1615 for 1 dpp and p = 0.5997; **Fig 3A** and **Fig 3B**). This suggests that MMTV-driven deletion of *Peg3* alone may not have any major impact on growth rates of the pups during gestation and also during postnatal stages.

The second set of breeding experiments were performed through crossing male *Peg3* ^FlpKO/WT^ with female Nkx2.1-Cre^+/tg^. The litter sizes at both 1 and 21-dpp time points were overall normal: 8 at 1 dpp and 7.8 at 21 dpp. The ratios of the pups with 4 genotypes were also close to the mendelian ratio of two independently segregating heterozygous loci (1:1:1:1) (p = 0.7698 for 1 dpp and p = 0.0649 for 21 dpp). The actual ratios for these breedings are shown in **Fig 3C and 3D**. This indicated no obvious lethality in the Nkx2.1-Cre/FlpKO pups from this breeding experiment. The weight profiles for the pups with these four genotypes also displayed no statistically significant variance at both time points, based on the one-way analysis of variance (ANOVA) (p = 0.593639 for 1 dpp and p = -0.643121 for 21 dpp). Collectively, the results from the two sets of breeding experiments concluded that MMTV-driven (mammary-specific) deletion of *Peg3* may produce lethality when litter size is constrained (as in MMTV-Cre first litters), but did not result in lethality from later litters. Also, MMTV-driven *Peg3* deletion did not affect growth rates. Our results also show Nkx2.1-driven (hypothalamus-specific) deletion of *Peg3* did not have any significant impact on survival and growth rates of the animals during gestation and postnatal stages.

### *Peg3* conditional knockout dams: milk letdown

**Fig 4:**
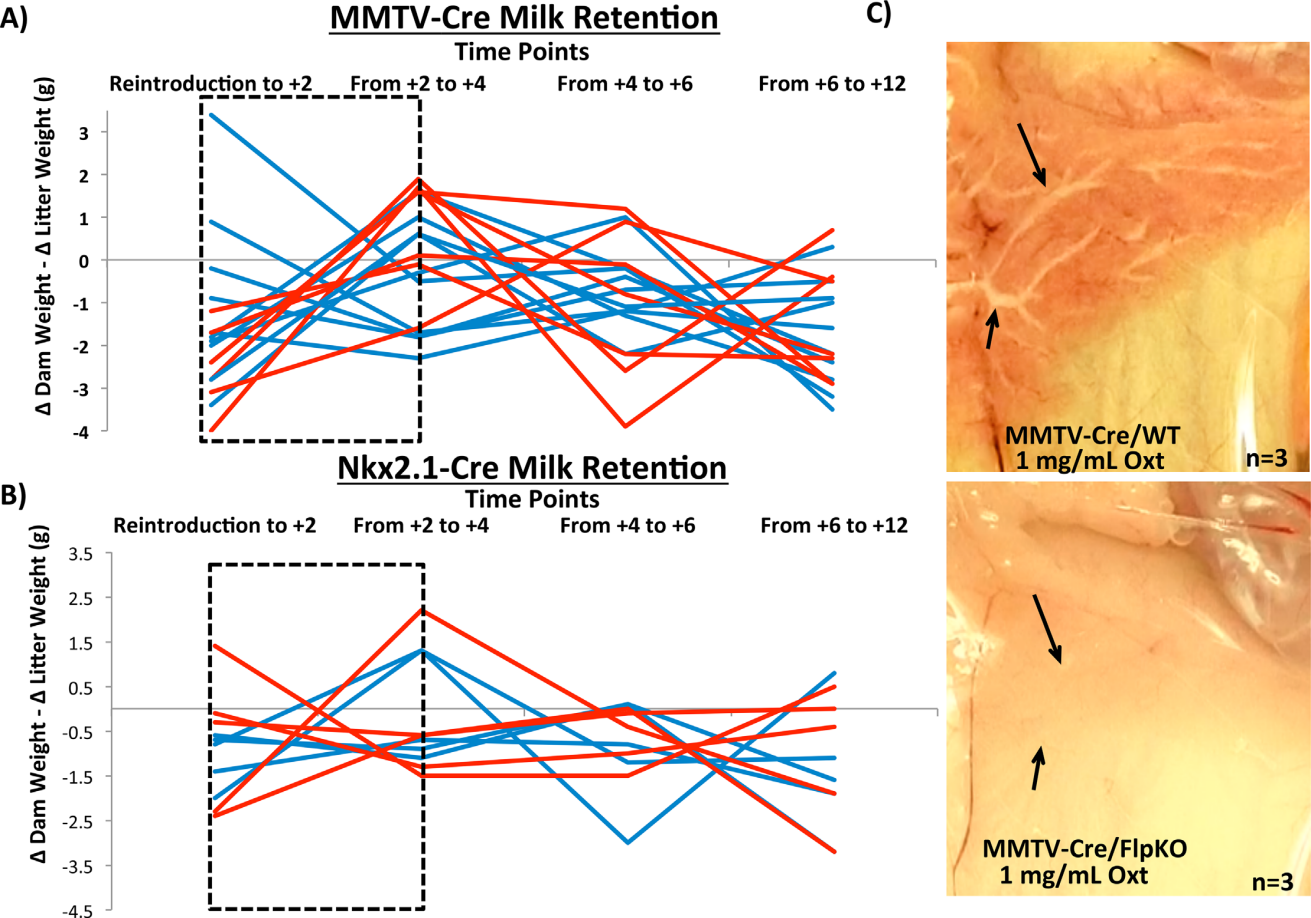
*Peg3* conditional knockout dams: milk letdown. Milk retention at 10 dpp of MMTV-driven **(a)** and Nkx2.1-driven **(b)** deletions in *Peg3* are shown as a function of the weight change between every two hours in the dam subtracted by the weight change between the same time points in the pups. Therefore, the weight flux shown is a measure of how much nutrient transfer is occurring from the dam to her pups at each given time interval with positive slopes indicating weight gain in the dam that is not transferred over to the pups. Conversely, negative slopes indicate a dam giving all her mass to the pups, without gaining any weight herself. The dashed black box indicates the critical point wherein reintroduction of the dams to their pups has just occurred. **(c)** Oxytocin-induced milk ejection was surveyed in MMTV-driven *Peg3* deleted mice alongside wild-type littermate controls. Black arrows indicate ductal branches in the mammary gland.

One of the mutant phenotypes associated with *Peg3* is a defect in milk letdown in nursing females [6,9]. This particular phenotype was detected among the females of the new mutant line *Peg3* ^WT/DelKO^ (data not shown). Four sets of females derived from the previous breeding experiments were further utilized to test this mutant phenotype of *Peg3*. This series of analyses used 7 MMTV-Cre/FlpKO, 7 MMTV-Cre/WT, 5 Nkx2.1-Cre/FlpKO and 5 Nkx2.1-Cre/WT mice. The test was performed in the following manner. The efficient milk letdown process by nursing females was evaluated through measuring weight (milk) transfer from nursing females to pups (**Fig 4A and 4B**). A nursing female was first separated from her litter for two hours, and then reintroduced to her pups. From the reintroduction (T0), the weights of the female and the litter were individually measured every two hours (**S4 Fig**). During every two-hour period, the weight change of the female (Δ Dam weight) was further subtracted by that of the litter (Δ Litter weight). For each female, a series of these subtracted weight values were summarized as a line graph to indicate the trend of the weight fluctuation. As shown in **Fig 4A**, the largest surge of weight (milk) transfer occurred during the Reintroduction to T+4 time period in the majority of the wild-type females (7 MMTV-Cre/WT, blue line). This is visualized by the blue lines with negative slope, indicating that the accumulated weight of nursing females was transferred to the weight of their litters. On the other hand, all of the mutant females (7 MMTV-Cre/FlpKO, red line) displayed positive slopes, indicating that the accumulated weight of the nursing females were not efficiently transferred to their pups during this time period. The slopes of the 10 dpp experiment was evaluated by t-test and the difference was shown to be statistically significant (p = 0.0137). This indicated that the mutant females lacking *Peg3* expression in the mammary gland most likely have a problem in releasing milk to their pups. This series of experiments was repeated with two litters for each female, and also performed at three different time points, at 5, 10, 15 dpp. Overall, the outcomes were consistent and reproducible among the different experiments. The results from the experiment at 10 dpp are shown as a representative set (**Fig 4A)**.

This series of experiments was also performed with another set of females (Nkx2.1-Cre/WT and Nkx2.1-Cre/FlpKO, **Fig 4B**). Comparison of the Nkx2.1-Cre/WT and Nkx2.1-Cre/FlpKO slopes by t-test did not show any significant difference between the two groups of females, indicating that the deletion of *Peg3* in the hypothalamus did not have any definitive impact on the weight (milk) transfer (p = 0.6915). To follow up on the results derived from these two series of experiments, we also performed an independent series of experiments testing the response of the mammary gland to the hormone oxytocin. For these experiments, three females from each of the four groups (MMTV-Cre/WT, MMTV-Cre/FlpKO, Nkx2.1-Cre/WT, Nkx2.1-Cre/FlpKO) were sacrificed, and their mammary glands were exposed to a high concentration of oxytocin 1 mg/mL (10x physiological concentration) (**Fig 4C**). The milk flow through the mammary ducts was easily detected among the MMTV-Cre/WT females. In contrast, the milk efflux was not obvious among the 3 MMTV-Cre/FlpKO females tested, indicating that the mammary gland without *Peg3* expression is not responsive to the high dose of oxytocin. This further suggests potential defects in the oxytocin circuitry as a main cause for the observed defect in the milk letdown process. We also performed the same series of oxytocin-induced milk ejection experiments with the set of Nkx2.1-Cre/WT and Nkx2.1-Cre/FlpKO (**S5 Fig**). However, in the Nkx2.1-Cre lines, there was no observable difference in milk flow between the wild type and conditional knockout females. This set of results is also consistent with the results derived from the weight transfer experiments described above, showing the defects only among the females with the deletion of *Peg3* in the mammary gland. In summary, the results demonstrated that conditional knockout of *Peg3* in the mammary gland causes a major defect in the milk letdown process, most likely through affecting the oxytocin circuitry. It is also important to note that this defect is more readily detectable through mutating *Peg3* in the mammary gland than in the hypothalamus.

### *Peg3* conditional knockout dams: nest building

**Fig 5:**
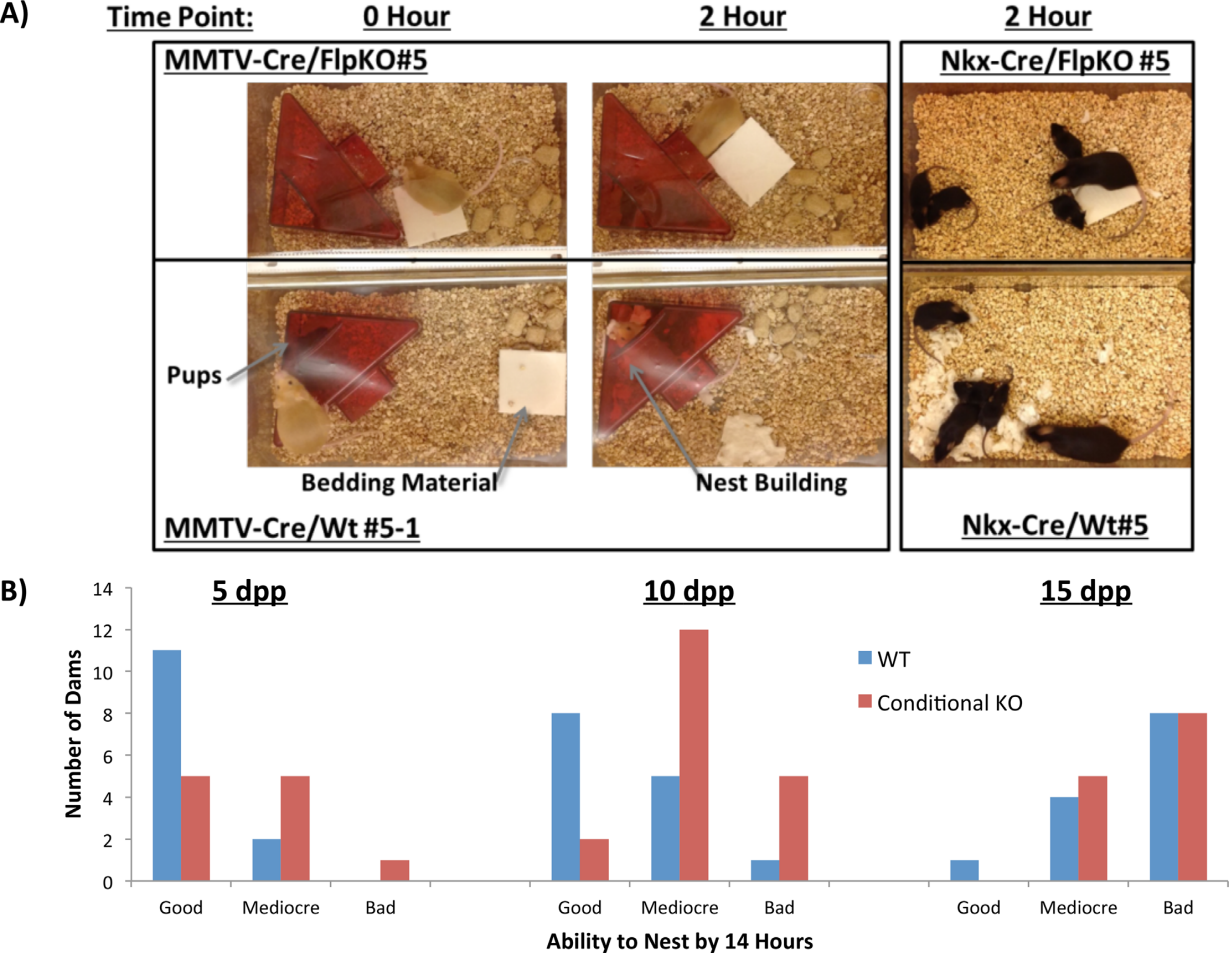
*Peg3* conditional knockout mothers: nest-building behavior. **(a)** Representative conditional knockout dams and wild-type littermates highlight differences observed in the nest-building behavior. **(b)** A graph shows the number of dams marked as good, mediocre and bad at building nests for their litters. “Good” dams are those who tore 100% of bedding material by 14 hours. “Mediocre” dams were able to use some, but not all of the bedding material by 14 hours. “Bad” dams are those that display no interest in tearing up the bedding material to insulate their pups.

While performing the weight transfer experiments, we also observed one behavioral abnormality that differentiated the wild type and mutant females, nest-building behavior. Nursing females usually tear apart solid bedding materials and build nests to provide insulation for their pups. However, both mutant types, MMTV-Cre/FlpKO and Nkx2.1-Cre/FlpKO, were found to be inefficient at nest building (**Fig 5A**). To systematically quantify this behavioral defect, we measured the weight of the untorn bedding materials every two hours. The relative weight of the remaining material to the initial weight was used to measure the efficiency of the nest-building behavior. The nursing females were further ranked from the best to worst nest builder based on their performance, which was judged by the relative weight of the remaining bedding materials (**Fig 5B, S1 Table, S2 Table** and **S3 Table**). According to the results, a large fraction of both mutant females, MMTV-Cre/FlpKO and Nkx2.1-Cre/FlpKO, were consistently ranked at the bottom of the list, thus making up the majority of the worst nest builders (**Fig 5B**, **S1 Table, S2 Table** and **S3 Table**). We are providing one short video demonstrating this behavioral problem (**S1 Movie**). We used the same set of nursing females and litters as the weight transfer experiment (**Fig 4A and 4B**), and also repeated two litters for each female at three different time points, 5, 10, 15 dpp. Overall, the outcomes were consistent and reproducible among individual sets of experiments. Regardless of genotype, we observed that the nest-building behavior of the animals was the most obvious during the early stage of nursing, at 5 dpp. Once the pups have fur (around 10 dpp), this behavior of the nursing female slowly diminished (**Fig 5B, S1 Table** and **S3 Table**). Nest building behavior was also the most obvious in the first litter for each female. T-tests between the WT and conditional knockout dams confirm the difference is statistically significant at 5 dpp (p = 0.0377, N = 24) and 10 dpp (p = 0.0053, N = 33), while this was not the case at 15 dpp (p = 0.7418, N = 26). In summation, the results from this series of experiments indicated that females with the mutation of *Peg3* in both the MMTV-driven and Nkx2.1-driven *Peg3* mutation have a problem in the nest-building behavior compared to their littermates. This nest-building behavior appears to be a subtle behavior of the mouse that is very closely associated with the dosage and functional sites of *Peg3*, since a deletion of *Peg3* in both MMTV and Nkx2.1-driven tissue affects the trait.

## Discussion

In the current study, two new mouse lines targeting the *Peg3* locus, *Peg3* ^FlpKO^ and *Peg3* ^DelKO^, have been derived and subsequently characterized at the molecular and organismal levels. The *Peg3* ^DelKO^ line, lacking Exon 6, displayed a set of phenotypes that are consistent with those observed from previous mutant alleles targeting *Peg3*. The *Peg3* ^FlpKO^ line, containing a floxed allele, was also utilized to further characterize the two well-known phenotypes of *Peg3*, a defect in the milk letdown process and in the nest-building behavior. According to the results, *Peg3*’s role in the mammary gland appears to be critical for the milk letdown process. In contrast, *Peg3*’s role in both the MMTV- and Nkx2.1-expressing tissues is important for the nest-building behavior. Overall, the current study introduced two additional mutant lines targeting *Peg3*, and also further characterized the mutant phenotypes of *Peg3* in terms of its tissue specific contribution.

The new mutant line *Peg3* ^DelKO^ displayed a similar set of phenotypes that have been seen from the other mutant alleles of *Peg3* (**Fig 2**). It is worthwhile to compare this new allele with the other existing mutant alleles of *Peg3*. This new mutant allele lacks only a small portion of the genomic region, a 600-bp region containing Exon 6 (**Fig 1A**), whereas the other alleles have an insertion of large expression cassettes in either Exon 5 or Intron 5 [6,9]. The mode of operation in these loss-of-function mutant alleles is also quite different. In the case of the *Peg3* ^DelKO^ allele, the transcription of *Peg3* is not affected at all, but the translation of the mutant transcript is affected, thus causing a complete abrogation of the PEG3 protein (**Fig 1D**). By contrast, the two existing models truncate the transcription of *Peg3* through the Poly-A signals that have been included as part of the expression cassettes. Thus, the transcription of *Peg3* is affected in both mutant models. This could have potential side effects since the transcription and/or transcript of an imprinted gene is known to influence the function of the adjacent genes. In that regard, it is important to note that the 3’-UTR of *Peg3* contains another imprinted gene, *APeg3* (Antisense *Peg3*) [15,16]. The transcriptional truncation of *Peg3* (sense gene) might have unknown consequences on the transcription and thus function of *APeg3* (antisense gene). Besides these differences, the two existing mutant models have an insertion of relatively large genomic fragments, spanning 5 to 7 kb in length, which might also have unknown effects on the locus itself and/or the adjacent imprinted genes. Nevertheless, all three models seem to have a common phenotype, delayed growth rate, although the newest model *Peg3* ^DelKO^ appears to have a more dramatic effect during the postnatal stage than during early embryogenesis (**Fig 2**). Given this observation, we can safely conclude that the observed phenotype is most likely caused by the lack of the gene product, the PEG3 protein, rather than the other defects potentially present in each of these mutant models. Furthermore, it is also important to note that the observed phenotype is also derived from mutant mice with the pure genetic background, C57BL/6, ruling out any possible complications that might originate from differing genetic backgrounds.

The *Peg3* ^FlpKO^ line was utilized for a series of conditional knockout experiments in pups and dams to further dissect *Peg3*’s role in neonatal development and the milk letdown process, respectively (**Figs 3 and 4**). According to the results, we were not able to produce the growth effects observed in *Peg3* mutant pups using conditional deletions. However, the use of conditional knockout dams allowed more insight into the tissue-specific roles of *Peg3*. The results indicate the mutation of *Peg3* in the mammary gland seems to provide a more readily detectable defect than the mutation in the hypothalamus. This is quite intriguing since it has been believed that the main cause for this defect may be due to the reduced number of oxytocin-producing neurons in the *Peg3* mutant mice [6]. Although we have not thoroughly analyzed this aspect of the mutant mice that have been used for the current study, it is difficult to envision that the MMTV-driven deletion of *Peg3* could have a drastic effect on the number of these specific neurons in the hypothalamus. Thus, we believe that this mutant phenotype of *Peg3* is most likely caused by a defect at a more global level than at a local level. One possibility would be that this might be caused by a lost connection in the oxytocin circuitry of the mutant mice, but not simply by a defect at one tissue. For the milk letdown process, the oxytocin circuitry requires the proper communication between the hypothalamus and the mammary gland through its molecular ligand and receptor. Any defect in this circuitry could easily cause a problem in the milk letdown process. It is important to note that *Peg3* is highly expressed in the tissues that are known to respond to the ligand, oxytocin, such as the ovary, uterus and placenta [2]. Thus, it is also possible that *Peg3* might be involved in the proper function of the receptor, oxytocin receptor. This possibility should be particularly interesting to pursue since *Peg3* has been recently identified as a DNA-binding transcription factor [4]. It is reasonable to predict that *Peg3* could modulate both the ligand and receptor for this circuitry. Thus, it would be of great interest to further narrow down the actual cause of this mutant phenotype in the near future.

The nest-building behavior of nursing females is shown to be affected by the mutation of *Peg3* in both MMTV- and Nkx2.1-driven lines (**Fig 5**). In both cases, the mutant females were either slow or inefficient at nest-building for their pups. This maternal caring defect is consistent with a previous *Peg3* knockout line that was characterized for maternal care phenotypes [6]. However, the data collected in this study also displays the limitations of these conditional knockout lines. First, inherent variability in the efficiency of Cre will result in a variable degree of *Peg3* deletion among different individuals (even among littermates with the same genotype, **S3 Fig, S1 Table, S2 Table** and **S3 Table**). Second, as the conditional deletions of *Peg3* rarely result in 100% deletion in the tissues targeted, this limits our ability to understand the full contribution of *Peg3* in these tissues. Despite these two limitations, these results help us understand the functions of *Peg3* in regards to expression sites and dosage, as two general patterns also emerge from our data. First, only the MMTV-driven deletion of *Peg3* impacted lactation, while either the Nkx2.1- or the MMTV-driven deletion of *Peg3* resulted in behavioral effects. This suggests that the behavioral phenotype is more sensitive to dosage changes than the more physiological, lactation phenotype. Second, there was no significant difference in growth of the *Peg3* conditional knockouts, yet effects on lactation and maternal care were still observed (**Figs 3–5**). This suggests the growth effects may require the mutation of *Peg3* at a more global level than the lactation and maternal care phenotypes. Overall, the results displayed a hierarchy of phenotypes in response to a loss of *Peg3*, the germline deletion exhibited all phenotypic effects, while both the MMTV and Nkx2.1-driven deletion presented nest-building effects and only the MMTV-driven deletion demonstrated lactation effects.

## Ethics Statement

All the experiments related to mice were performed in accordance with National Institutes of Health guidelines for care and use of animals, and also approved by the Louisiana State University Institutional Animal Care and Use Committee (IACUC), protocol #13–061.

## Materials and Methods

### Generation of *Peg3* ^FlpKO^ and *Peg3* ^DelKO^ strains

The *Peg3* ^CoKO^ strain has been generated using a targeted ES cell, *Peg3* ^tm1a (EUCOMM) Hmgu^, from the EUCOMM (European Conditional Mouse Mutagenesis) consortium, and has been maintained in the lab through maternal transmission for multiple generations [9]. The *Peg3* ^FlpKO^ line has been derived by crossing *Peg3* ^CoKO^ with the commercially available Rosa26-FLP line (Jackson Lab, Stock No. 009086, B6.129S4-*Gt* (ROSA)*26Sor* ^*tm1(FLP1)Dym*^/RainJ). Progeny were tested for the excision of β-Galactosidase and Neomycin resistance markers by PCR using the set of the following primers: *Peg3*-5ARM (5’-CCCTCAGCAGAGCTGTTTCCTGCC-3’) and *Peg3*-3ARM (5’-AAGCTACCTGGGAAATGAGTGTGG-3’). These generated mice were further screened to remove the Rosa26 Flippase allele by PCR using the following set of primers: NEW-ROSA26-FlippaseF (5’-TAAGTGAGGGTGAAAGCATCTGGG-3’) and NEW-ROSA26 FlippaseR (5’-ACTCGTTTTAGGACTGGTTCAGA-3’). The identified mice heterozygous for the *Peg3* ^FlpKO^ allele without the ROSA26-Flippase allele have been maintained with the mutant allele being transmitted as a maternal allele. This *Peg3* ^FlpKO^ line has been maintained through backcrossing with the C57BL/6J strain for greater than 5 generations. Primers used for confirming the intact *Peg3*WT/FlpKO and *Peg3*WT/DelKO transcripts (S1 Fig) are as follows: For exon1-3 amplification: *Peg3*-RT-1a (5’-GGTTCAGTGTGGGTGCACTAGACT-3’), *Peg3*-RT-1b.1: (5’-GCTCACACCCAAGGGCTTGAGCGT-3’); for Exon 1–7 amplification: *Peg3*-RT-1a (5’-GGTTCAGTGTGGGTGCACTAGACT-3’), *Peg3*-RT-1b.2 (5’-CTGAGGCTTCTTGTCCTCTTTGAG-3’); for exon 1–9 amplification: *Peg3*-RT-1a (5’-GGTTCAGTGTGGGTGCACTAGACT-3’), *Peg3*-RT-1b.3 (5’-TCCCTAGTGTGCATGATCTGGT-3’).

For conditional knockout experiments, the *Peg3* ^FlpKO^ mouse line was then crossed with commercially available MMTV-Cre (Stock # 003553, B6129-Tgn(MMTV-Cre)4Mam-LineD) and Nkx2.1-Cre (Stock # 008661 C57BL/6J-Tg(Nkx2-1-cre)2Sand/J) lines from Jackson lab[17,18]. MMTV-Cre line D was used for its restricted expression in the brain (<10% recombination) and its inability to affect mammary development [19]. Primers used to test recombination in tissues from the conditional knockout breeding are: Pre-LoxF(5’-TGGACTTGGGAGATACTGTATTTATCAGCA-3’), LoxR1(5’-TGCCTTCTCTTTAGAGTGACTGAGG-3’) and PostLoxR2 (TCTGACTTCCTGGGAGCCAGTAAGA-3’).

The *Peg3* ^DelKO^ line has been derived through the crossing of female *Peg3* ^WT/FlpKO^ with male Zp3-Cre (Jackson Lab, Stock No. 003651, C57BL/6-Tg (Zp3-cre) 93Knw/J). Female heterogygous for the *Peg3* ^DelKO^ allele were screened with the following set of primers: *Peg3*-5ARM and *Peg3*-LoxR (5’- TGAACTGATGGCGAGCTCAGACC-3’). These F1 mice were further tested for the absence of the Cre recombinase cassette by PCR with the following set of primers: Zp3-CreF (5’-TAGGAATCACGTGGAGTGTCT-3’) and oIMR1085 (5’-GTGAAACAGCATTGCTGTCACTT-3’). This *Peg3* ^DelKO^ line was similarly backcrossed with male C57BL/6J for greater than 4 generations. These mice were then used for testing the neonatal growth experiments. For genotyping, ear snips were incubated overnight in the lysis buffer (50 mM Tris-Cl at pH 8.0, 100 mM EDTA at pH 8.0, 250 mM NaCl, 1% SDS, along with 20 μg/mL Proteinase K) at 65°C. The 60-fold diluents of these lysed samples were used as a temple for PCR amplification using the PCR premix kit (iNtRON Biotech) at the following conditions (Step 1, 95°C-30 sec; Step 2, 95°C-30 sec, 60°C-30 sec, 72°C-60 sec for 35 cycles; Step 3, 72°C-10 min).

### DNA isolation from tissues

The DNA isolation from placenta, mammary gland and hypothalamus (**S4 Fig**) were performed similarly to the genotyping protocol above, but the addition of a phenol-chloroform extraction was necessary. Mice were sacrificed using CO_2_, the tissues were excised from the mouse, then immediately placed into lysis buffer with 20μg/mL Proteinase K and incubated at 65°C. Once lysed, the samples were purified using phenol-chloroform extraction, then used for PCR amplification using the PCR premix kit (iNtRON Biotech) at the following conditions (Step 1, 95°C-30 sec; Step 2, 95°C-30 sec, 60°C-30 sec, 72°C-60 sec for 35 cycles; Step 3, 72°C-10 min)

### Milk retention and maternal care experiments

As displayed in the conditional knockout breeding scheme (**S2 Fig**), four different types of females were bred with male B6 to observe maternal care and lactation phenotypes. Beginning one hour after the start of the dark cycle, dams, their litters and bedding material were weighed and pups were separated from their dams. Pup/mother separation lasted for 2 hours and was followed by weight measurements of the same materials and a reintroduction to the mother. Every two hours after reintroduction to the mothers, their litters and bedding material were monitored for weight gain/loss as well as whether or not the mother was crouching over the pups at the time of observation. This experimental setup was tested on 2 litters for each dam (5 Nkx-Cre/Wt, 5 Nkx-Cre/FlpKO, 7 MMTV-Cre/Wt, 7 MMTV-Cre/FlpKO) at three different time points (5 dpp, 10 dpp, and 15 dpp). To measure the efficiency of nutrient transfer from the dams to the pups, we subtracted the amount of weight gain/loss of the litter from the amount of gain/loss of the dam every two hours. The inclusion and integration of the dam’s weight in these analyses differs from previous experiments where only the weight of the nursing litter was taken into account^7^. By incorporating the fluctuations in the dams’ weight, the analysis can better account for uncertainties brought about by changes in the eating behavior of the dams. Comparisons between WT and FlpKO dams could be used for analysis in this study. However, due to differences in genetic background, it is inadvisable to cross-compare the WT and FlpKO dams from MMTV-Cre and Nkx2.1-Cre dams using this approach.

The nest-building behavior was monitored with a modified protocol [20]. Modifications to the original protocol are as follows: 1) The weight of the bedding material was measured every two hours to determine the dams with exceptional nest-building behavior. 2) The time course was extended from 30 minutes to 14 hours to account for any dams with slow nest-building behavior. 3) The nest-building behavior was tested at three developmental stages: 5 dpp, 10 dpp and 15 dpp.

### Mammary oxytocin response experiments

Oxytocin response experiments were completed using the modified protocol [21]. We determined the optimal period of latency for the Nkx2.1-Cre mice to be 40 minutes, and for the MMTV-Cre mice to be 20 minutes to build up milk. Following CO2-mediated sacrifice of the conditional knockout and wild-type dams, mammary glands were exposed. Pictures of the mammary glands were taken prior to addition of any solutions. Phosphate-buffered saline (PBS) was added to both sides of the mouse mammaries, then syphoned off and the mammaries were imaged. Then, Oxytocin (Sigma Cat No O4375), re-suspended in 1x PBS at a concentration of 1 mg/mL, was added to the mammary and allowed to incubate for 1 minute before the solution was syphoned off. The mammary was then imaged. This protocol was performed for three of each conditional knockout and corresponding WT littermates.

### cDNA synthesis and Reverse-Transcription PCR (RT-PCR)

Total RNA was isolated from mouse organs using Trizol (Life Technologies Cat No 10296028) according to manufacturer’s protocol. The isolated RNA was first reverse-transcribed using the M-MLVRT First-Strand Synthesis System (Invitrogen Cat No. 28025–013) in accordance with manufacturer’s protocol with random hexamer and oligo-dT primers. Subsequent cDNA was used as a template for RT-PCR. All RT-PCR reactions were carried out for 33 cycles under standard PCR conditions. The primers for *Peg3* and *β-Actin* were as follows: *Peg3*-RT-Exon3F2 (5’-ATCCCTGAAACGCTCAAGCCCT-3’), *Peg3*-Ex7R (5’-CTCCAGGTTGTCCTGAATTGGA-3’), βactin-RT1a (5’-GAGCACCCTGTGCTGCTCACCGA-3’) and βactin-RT1b (5’- CTCTTTGATGTCACGCACGATTTC-3’).

### Protein isolations and western blots

Mice were sacrificed by cervical dislocation in accordance with IUCAC guidelines. Whole heads were harvested and immediately homogenized in lysis buffer (0.25M Tris–HCl, pH 7.8, plus 0.1% NP-40) with Proteinase Inhibitor Cocktail (EMD-Millipore Cat No 539131) at 1x concentration. The cellular debris was removed by centrifugation for 10 minutes at 4°C. Protein concentrations were determined by the Bradford assay kit (Pierce), using diluted BSA as protein standards. Sixty micrograms of each lysate were separated on 10% SDS–PAGE gels and transferred to PVDF membranes (Hybond-P, Amersham) using a Mini Trans-Blot wet transfer cell (Bio-Rad). Membranes were blocked for 1 hour in the Tris-buffered saline (TBS) containing 1% skim milk and 0.05% Tween 20, and incubated overnight at 4°C with a custom-made purified anti-PEG3 antibody [9]. These blots were incubated for an additional 1 hour with the secondary antibody linked to horseradish peroxidase (Sigma Cat No A6154). The blots were developed using the Western blot detection system according to the manufacturer’s protocol (Thermo Scientific Cat No 17295).

## Supporting Information

S1 Fig RT-PCR full-length *Peg3* transcipt. RNA was extracted and RT-PCR was performed on brain samples from *Peg3* ^WT/WT^ and a) *Peg3* ^WT/FlpKO^ and b) *Peg3* ^WT/DelKO^ adult mouse brain. Resulting cDNA was amplified using different primer combinations to amplify from exon 1 to exon 3 (lanes 1 and 2), from exon 1 to exon 7 (lanes 3 and 4) and from exon 1 to exon 9 (lanes 5 and 6). *Peg3* ^WT/WT^ samples are in the odd-numbered lanes (lanes 1,3 and 5), while *Peg3* ^WT/FlpKO^ samples are in the even-numbered lanes (lanes 2, 4 and 6). The minor band observed in lane 4 of the Peg3^WT/DelKO^ was sequenced and determined to be a minor splice variant skipping exons 3–6. Amplicon sizes are noted next to the gel image.
(TIFF)

S2 Fig Breeding scheme for conditional knockouts. **(Cross A)** *Peg3* ^FlpKO^ males were crossed with Nkx2.1-Cre females to produce offspring with paternally-transmitted deletions of *Peg3*. These pups were tested for lethality and growth defects that resulted in **Fig 3C and 3D**. Female Nkx2.1-Cre/WT and Nkx2.1-Cre/FlpKO from these experiments were then used for **(Cross B)**, where they were bred with B6 males to be used for testing lactation and maternal caring behaviors. A similar breeding scheme was used for **(Cross C)** and **(Cross D)**. However, MMTV-Cre females in **(Cross C)** were available as MMTV-Cre^tg/tg^, which allowed for a simpler breeding scheme.
(TIFF)

S3 Fig *Peg3* recombination in tissues correlated with growth and lactation phenotype. Genomic DNA was isolated from tissues known to have high expression of *Peg3* and have implications in reproduction. Mammary and hypothalamus were isolated from dams who inherited the *Peg3* ^FlpKO^ allele, along with the conditionally-expressing Cre line indicated. Placentas were isolated from earlier experiments, wherein the embryos inherited the *Peg3* ^FlpKO^ allele and the MMTV-driven Cre allele. The *Peg3* ^FlpKO^ and *Peg3* ^DelKO^ alleles were amplified from the mammary and placenta using (5-ARM, *Peg3*-LoxR primers) and are indicated by the red labels with arrows (a,b). Hypothalamic regions and mammary glands were amplified using (PreLoxF, LoxR1 and PostLoxR2 primers) in a 3-primer combination, which display the *Peg3* ^WT^,*Peg3* ^FlpKO^ and *Peg3* ^DelKO^ alleles.
(TIFF)

S4 Fig Overnight lactation and nest-building experiment scheme. This cartoon displays the overall schematic for milk provision and nest-building behavior experiments.
(TIFF)

S5 Fig Oxytocin-induced milk ejection in Nkx2.1-Cre dams. Similar to the experiment observed in **Fig 4C**, 1 mg/mL oxytocin in 1x PBS was dripped onto the mammary glands of an Nkx2.1-Cre/FlpKO dam and WT littermate. Mammary glands were then visualized for movement of milk through the mammary ducts.
(TIFF)

S1 Movie *Peg3* conditional knockout nest-building defect. The video shows maternal caring behavior in a representative MMTV-driven *Peg3* knockout dam and her WT littermate. As seen in **Fig 5A**, the 5-dpp litters are stationed in red plastic houses and provided with a square of bedding material. Video was taken over a 3-hour period and then time-lapsed to 30 seconds. A time lapse of the 3 hour video shows that the WT dam successfully insulates her litter by tearing 100% of the bedding material, while the MMTV-Cre/FlpKO littermate does not display the nest-building behavior.
(MOV)

S1 Table Raw nesting-building behavior data from the pups at 5dpp. These tables display the percent of the original bedding material that was still left at each time point from all four genotypes tested (MMTV-Cre/WT, MMTV-Cre/FlpKO, Nkx2.1-Cre/WT, Nkx2.1-Cre/FlpKO). The data displayed in their corresponding tables are as follows: 5 dpp age groups are in **S1 Table**. 10 dpp are in **S2 Table**. 15 dpp are in **S3 Table**. Dams with WT genotypes are in black, while dams harboring the FlpKO allele are in Red. Red dashed lines indicate borders used to separate the “Good”, “Mediocre” and “Bad” nesting behaviors in **Fig 5B**.
(TIFF)

S2 Table Raw nesting-building behavior data from the pups at 10dpp. These tables display the percent of the original bedding material that was still left at each time point from all four genotypes tested (MMTV-Cre/WT, MMTV-Cre/FlpKO, Nkx2.1-Cre/WT, Nkx2.1-Cre/FlpKO). The data displayed in their corresponding tables are as follows: 5 dpp age groups are in **S1 Table**. 10 dpp are in **S2 Table**. 15 dpp are in **S3 Table**. Dams with WT genotypes are in black, while dams harboring the FlpKO allele are in Red. Red dashed lines indicate borders used to separate the “Good”, “Mediocre” and “Bad” nesting behaviors in **Fig 5B**.
(TIFF)

S3 Table Raw nesting-building behavior data from the pups at 15dpp. These tables display the percent of the original bedding material that was still left at each time point from all four genotypes tested (MMTV-Cre/WT, MMTV-Cre/FlpKO, Nkx2.1-Cre/WT, Nkx2.1-Cre/FlpKO). The data displayed in their corresponding tables are as follows: 5 dpp age groups are in **S1 Table**. 10 dpp are in **S2 Table**. 15 dpp are in **S3 Table**. Dams with WT genotypes are in black, while dams harboring the FlpKO allele are in Red. Red dashed lines indicate borders used to separate the “Good”, “Mediocre” and “Bad” nesting behaviors in **Fig 5B**.
(TIFF)
