# Supplementary figures and images for: Tissue-Specific Contributions of Paternally Expressed Gene 3 in Lactation and Maternal Care of Mus musculus
Source: PLoS One. 2015 Dec 7;10(12):e0144459. doi: 10.1371/journal.pone.0144459 (PMC4671625; doi:10.1371/journal.pone.0144459)

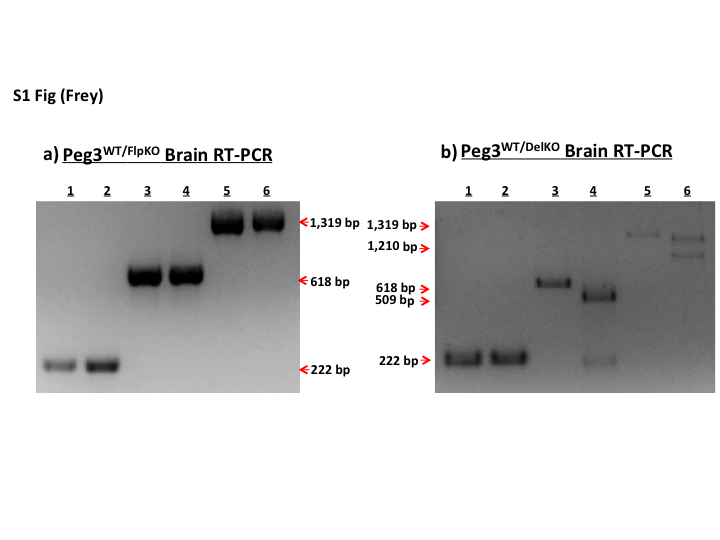

Supplement: S1 Fig — RNA was extracted and RT-PCR was performed on brain samples from Peg3 WT/WT and a) Peg3 WT/FlpKO and b) Peg3 WT/DelKO adult mouse brain. Resulting cDNA was amplified using different primer combinations to amplify from exon 1 to exon 3 (lanes 1 and 2), from exon 1 to exon 7 (lanes 3 and 4) and from exon 1 to exon 9 (lanes 5 and 6). Peg3 WT/WT samples are in the odd-numbered lanes (lanes 1,3 and 5), while Peg3 WT/FlpKO samples are in the even-numbered lanes (lanes 2, 4 and 6). The minor band observed in lane 4 of the Peg3WT/DelKO was sequenced and determined to be a minor splice variant skipping exons 3–6. Amplicon sizes are noted next to the gel image. (TIFF) [file pone.0144459.s001.tiff]

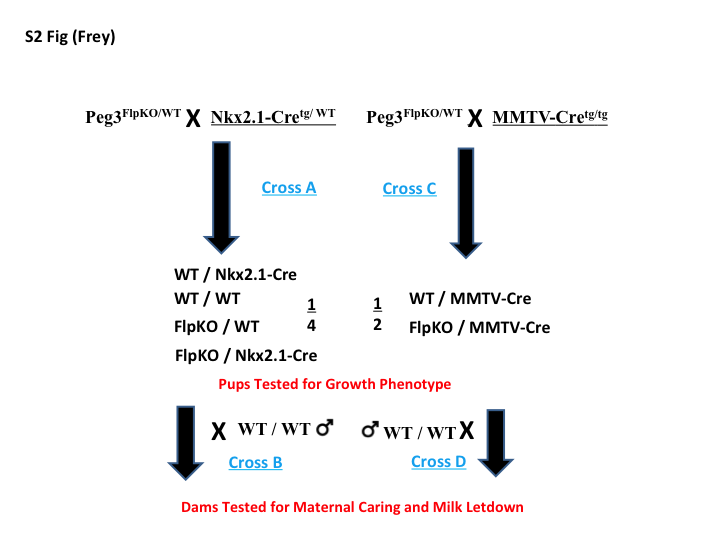

Supplement: S2 Fig — (Cross A) Peg3 FlpKO males were crossed with Nkx2.1-Cre females to produce offspring with paternally-transmitted deletions of Peg3. These pups were tested for lethality and growth defects that resulted in Fig 3C and 3D. Female Nkx2.1-Cre/WT and Nkx2.1-Cre/FlpKO from these experiments were then used for (Cross B), where they were bred with B6 males to be used for testing lactation and maternal caring behaviors. A similar breeding scheme was used for (Cross C) and (Cross D). However, MMTV-Cre females in (Cross C) were available as MMTV-Cretg/tg, which allowed for a simpler breeding scheme. (TIFF) [file pone.0144459.s002.tiff]

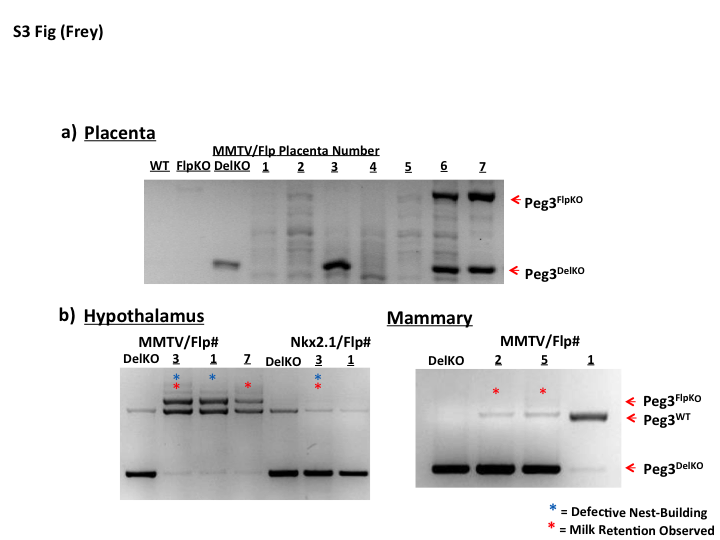

Supplement: S3 Fig — Genomic DNA was isolated from tissues known to have high expression of Peg3 and have implications in reproduction. Mammary and hypothalamus were isolated from dams who inherited the Peg3 FlpKO allele, along with the conditionally-expressing Cre line indicated. Placentas were isolated from earlier experiments, wherein the embryos inherited the Peg3 FlpKO allele and the MMTV-driven Cre allele. The Peg3 FlpKO and Peg3 DelKO alleles were amplified from the mammary and placenta using (5-ARM, Peg3-LoxR primers) and are indicated by the red labels with arrows (a,b). Hypothalamic regions and mammary glands were amplified using (PreLoxF, LoxR1 and PostLoxR2 primers) in a 3-primer combination, which display the Peg3 WT,Peg3 FlpKO and Peg3 DelKO alleles. (TIFF) [file pone.0144459.s003.tiff]

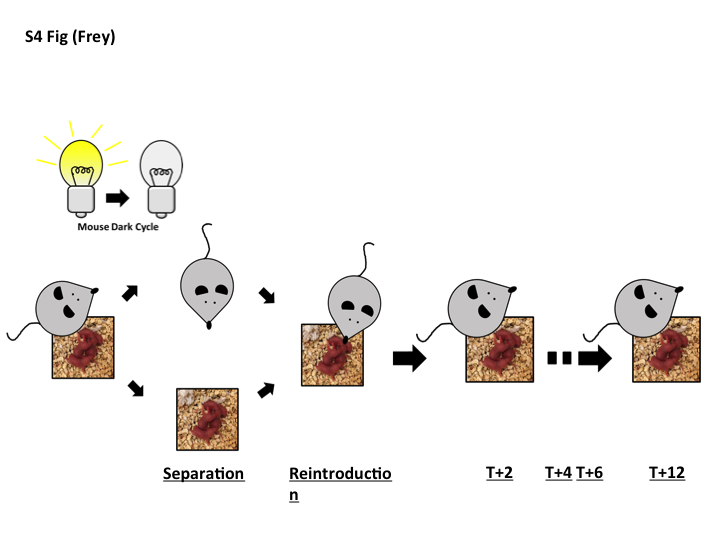

Supplement: S4 Fig — This cartoon displays the overall schematic for milk provision and nest-building behavior experiments. (TIFF) [file pone.0144459.s004.tiff]

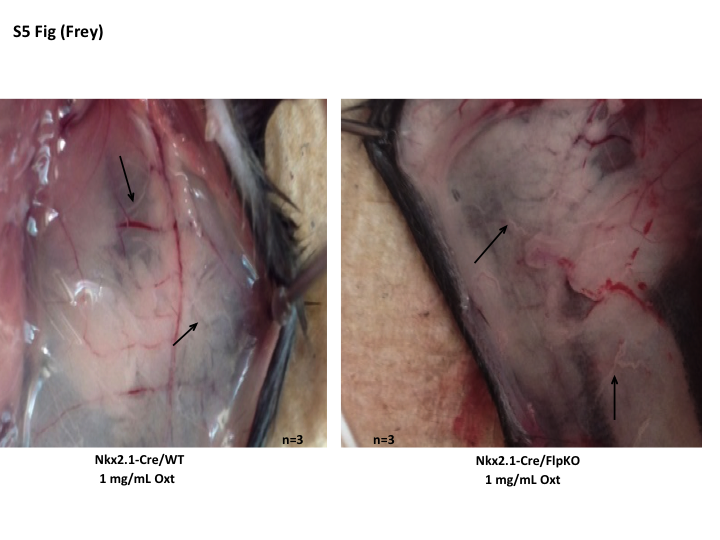

Supplement: S5 Fig — Similar to the experiment observed in Fig 4C, 1 mg/mL oxytocin in 1x PBS was dripped onto the mammary glands of an Nkx2.1-Cre/FlpKO dam and WT littermate. Mammary glands were then visualized for movement of milk through the mammary ducts. (TIFF) [file pone.0144459.s005.tiff]

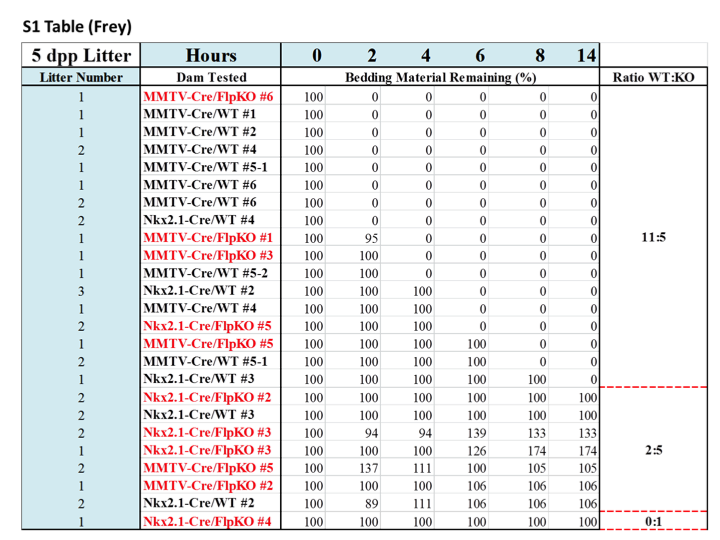

Supplement: S1 Table — These tables display the percent of the original bedding material that was still left at each time point from all four genotypes tested (MMTV-Cre/WT, MMTV-Cre/FlpKO, Nkx2.1-Cre/WT, Nkx2.1-Cre/FlpKO). The data displayed in their corresponding tables are as follows: 5 dpp age groups are in S1 Table. 10 dpp are in S2 Table. 15 dpp are in S3 Table. Dams with WT genotypes are in black, while dams harboring the FlpKO allele are in Red. Red dashed lines indicate borders used to separate the “Good”, “Mediocre” and “Bad” nesting behaviors in Fig 5B. (TIFF) [file pone.0144459.s007.tiff]

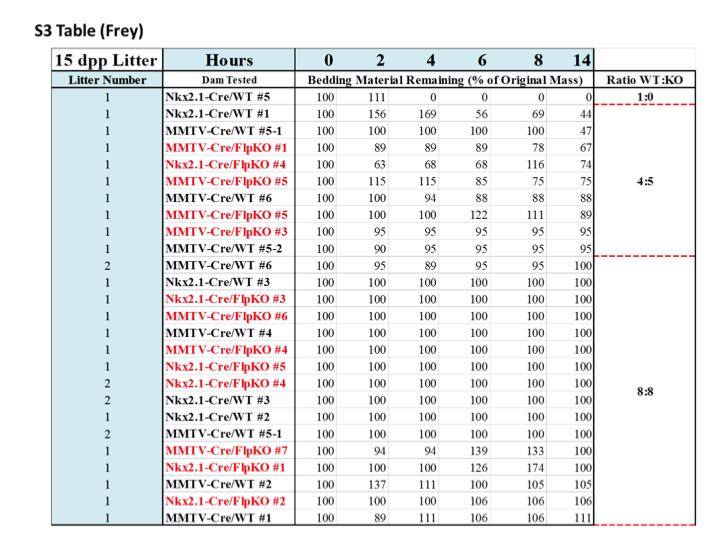

Supplement: S2 Table — These tables display the percent of the original bedding material that was still left at each time point from all four genotypes tested (MMTV-Cre/WT, MMTV-Cre/FlpKO, Nkx2.1-Cre/WT, Nkx2.1-Cre/FlpKO). The data displayed in their corresponding tables are as follows: 5 dpp age groups are in S1 Table. 10 dpp are in S2 Table. 15 dpp are in S3 Table. Dams with WT genotypes are in black, while dams harboring the FlpKO allele are in Red. Red dashed lines indicate borders used to separate the “Good”, “Mediocre” and “Bad” nesting behaviors in Fig 5B. (TIFF) [file pone.0144459.s008.tiff]

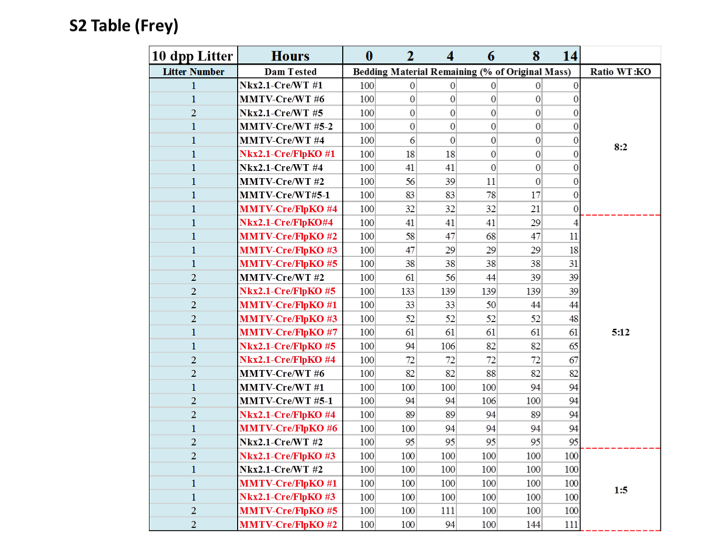

Supplement: S3 Table — These tables display the percent of the original bedding material that was still left at each time point from all four genotypes tested (MMTV-Cre/WT, MMTV-Cre/FlpKO, Nkx2.1-Cre/WT, Nkx2.1-Cre/FlpKO). The data displayed in their corresponding tables are as follows: 5 dpp age groups are in S1 Table. 10 dpp are in S2 Table. 15 dpp are in S3 Table. Dams with WT genotypes are in black, while dams harboring the FlpKO allele are in Red. Red dashed lines indicate borders used to separate the “Good”, “Mediocre” and “Bad” nesting behaviors in Fig 5B. (TIFF) [file pone.0144459.s009.tiff]
